# Thermodynamic Modeling and Experimental Validation of Acetic Acid Attack on Hardened Cement Paste: Effect of Silica Fume

**DOI:** 10.3390/ma15238355

**Published:** 2022-11-24

**Authors:** Felix Berger, Andreas Bogner, Astrid Hirsch, Neven Ukrainczyk, Frank Dehn, Eduardus Koenders

**Affiliations:** 1 Institute of Construction and Building Materials, Technical University of Darmstadt, 64287 Darmstadt, Germany; 2 Institute of Concrete Structures and Building Materials, Department Building Materials and Concrete Construction, Karlsruhe Institute of Technology (KIT), 76131 Karlsruhe, Germany

**Keywords:** cementitious materials, acid attack, silica fume, pozzolanic reaction, thermodynamic modeling, IPHREEQC, solid-liquid phase equilibration experiments

## Abstract

Concrete structures are increasingly becoming exposed to organic acid attack conditions, such as those found in agriculture and food-related industries. This paper aims to experimentally verify the thermodynamic modeling of cement pastes under acetic acid attack. For this, a modeling approach implemented in IPHREEQC via Matlab is described, and results are compared with measured pH and compositions of equilibrated solutions (MP-AES) as well as unreacted/precipitated solids (XRF, XRD and STA) for a wide range of acid concentrations. The 11% replacement of cement by silica fume (SF) led to a 60 or 70% reduction (measured or modeled, respectively) of Portlandite content in the hardened cement paste due to the pozzolanic reaction resulting in higher content of CSH phases, which has effects on the progression of dissolution processes and a resulting pH with increased acid concentrations. Considering that no fitting parameter was used, the model predictions showed good agreement with measured values of pH, dissolved ion concentrations and composition of the remaining (degraded) solids overall. The discrepancies here were more pronounced at very high acid concentrations (equilibrium pH < ~4), i.e., after the full dissolution of hydrate phases due to limitations in the model used to describe Al-, Si- and Fe-gel phases and/or identified experimental challenges in precipitation of calcium and aluminum acetate hydrates.

## 1. Introduction

In many situations, the resistance against acid attack is of vital importance for the durability of concrete. Structures in agricultural, agro-food and biogas plants are often exposed to organic acids, such as acetic, citric or lactic acid, which attack the concrete with different intensities [1]. Here, the solubility of acid salts formed during the degradation of the concrete matrix is an important factor to consider [2]. Acetic acid was chosen for this study as it has an intermediate aggressiveness in decalcifying and dissolving almost all hydrated and anhydrous phases in the cement matrix. Thereby acetic acid does not form expansive salts as the even more corrosive citric acid [2].

The type of binder also has a significant influence on the extent of deterioration of a cement paste matrix under acid attack. Concrete made of ordinary Portland cement (PC) usually has a lower acid resistance compared to concrete made of calcium aluminate cement (CAC) or mixtures of Portland cement and supplementary cementing materials (SCMs). The pozzolanic additives with high silicate (such as silica fume) and aluminate content (such as slag, metakaolin and fly ash) lead to a lower concentration of Portlandite in the hydrated paste and densification of the microstructure, both increasing the acid resistance [3].

For PC exposed to acetic acid, the deterioration process begins with the dissolution of Portlandite from the cement matrix. With a further attack of protons from the acid species, other hydration products, such as the calcium silicate hydrate (CSH) phases, begin to decalcify, ultimately resulting in an amorphous silica gel with small amounts of aluminum and iron [1]. Beneath the completely decalcified layer of silica gel, a partially degraded layer forms that are characterized by Portlandite dissolution only. This leads to a gradient of the pH with decreasing values from this layer towards the outer layer or the concrete surface [3].

During the dissolution and decalcification process, the acetate ions form relatively weak complexes with calcium, aluminum and iron (II) ions, as well as stronger complexes with iron (III) ions [3]. Precipitated ferric hydroxide appears as a characteristic light-brown discoloration at the deterioration front. The formation of a deteriorated layer, combined with the precipitation of acid salts and other secondary phases within this deteriorated layer, leads to an increasingly protective layer on the surface that can slow down the acid attack by limiting the amount of acid reaching the undamaged core [4].

Modeling acid attack of cementitious materials is a challenging task due to the multi-component and multi-scale porous nature of cementitious materials [5,6,7]. A fundamental modeling approach that couples the chemistry is needed, i.e., reaction thermodynamics and kinetics [8,9,10] and transport phenomena with a morphological nature of the diffusion coefficient (e.g., porosity effects) [11] and multi-scale distribution of mineral phases [12]. To avoid enormous computational costs arising from an explicit description of heterogeneities, only homogenized (averaged) materials models are used, neglecting heterogeneity descriptions and fundamentals occurring across a range of scales. They rely on measurements of effective (overall lumped) parameters describing transport (e.g., effective diffusion coefficient), reaction rate and neutralization capacity, each of which results from lumping the many underlying fundamental processes. First, the thermodynamic description needs to be well-understood before considering the kinetics effects. The chemistry description requires equilibrium (thermodynamics), neutralization capacity and dissolution rates of individual phases, as well as the effect of different types and concentrations of acids. Recent reactive transport models consider a thermodynamic (TD) approach for describing the multiple homogenous and heterogeneous equilibria behavior [8,9,13].

Usually, only selected acid concentrations or resulting pH values are investigated for acid exposed concretes. However, understanding the influence of different acid concentrations on the deterioration of hydrated cement paste is of key importance. Therefore, in this study, samples of hydrated cement paste were crushed and mixed into solutions of water and different amounts of acetic acid. The point at which equilibrium was reached was determined based on pH measurements. Afterward, the composition of the solution and potentially remaining solids were analyzed. To evaluate the ongoing reactions during an acid attack on cementitious materials or even to calculate material mass balance flows, it is not enough to focus on the altered cementitious samples only, but an additional chemical analysis of the fluid attack medium is crucial [14,15].

In this paper, the thermodynamic modeling of acidification and leaching of a realistic cement system was conducted in a newly developed Matlab code that links IPHREEQC with a recent TD database for cement hydration [16] and acetic acid solution [3,8]. The leaching and acidification cycle consisted of closely simulating the batch experimental setups by adjusting the aggressive solution to hydrated solids ratios for thermodynamic (re-equilibration) calculations. The modeling approach is described in detail. The batch reaction model was developed to enable better estimation of the effects of different acids, their concentrations or even other hydrated paste compositions. The model was split into two sections. In the first step, the cement composition and water/cement ratio used in the experiments were used as input parameters for hydration calculations. In the second step, the resulting solid composition of the hydrated paste was exposed to solutions of water and the different concentrations of acetic acid, which is also used in the experimental part. The equilibrium results of the model are compared to the results of the batch reaction experiments to validate the model. The results of this validation provided improved input parameters for reactive transport models, which can be used to improve the accuracy of service-life predictions. These optimized predictions will help in the engineering process for designing, monitoring and maintaining concrete structures exposed to acid attacks.

## 2. Materials and Methods

### 2.1. Materials

Two series of hardened cement paste (hcp) with a water-to-cement mass ratio (*w*/*c*) of 0.35 were investigated. While the first sample series (labeled PC) consisted of only deionized water and an ordinary Portland cement (PC) CEM I 42.5 R according to EN 197-1 obtained by HeidelbergCement AG (Heidelberg, Germany). For the second series (labeled PC + SF), 11 mass percent (maximum fraction according to EN 1045) of this cement was replaced by the silica fume powder (SF, also known as microsilica) Elkem Microsilica 940E, provided by Elkem ASA Silicon Materials (Trondheim, Norway). For PC + SF, the superplasticizer ViscoCrete-2014 (Sika Deutschland GmbH, Stuttgart, Germany) was added to equal 0.38% of the solid mass in order to adjust the spreading flow to one of the mixtures of PC. In each case, after mixing the solids and water with an IKA RE-166 stirrer (IKA-Werke GmbH & Co. KG, Staufen, Germany) at room temperature, a plastic bottle with a volume of one liter was completely filled with the resulting paste across three steps, always followed by densifying using a vibrating table (VT 355/560-C, Tonindustrie, Berlin, Germany). The sealed bottles were shaken for 24 h at room temperature in a Hei-MIX Reax 2 overhead shaker (Heidolph Instruments GmbH & Co. KG, Schwabach, Germany) to ensure a homogenous hardening and afterward stored at 20 °C. After 21 days, the hardened paste was removed from the bottle and stored in a small volume of deionized water at 20 °C. After 27 days in the case of PC and seven months in the case of PC + SF, the pastes were dried overnight at room temperature conditions, then ground with a vibrating disc mill (Pulverisette, Fritsch GmbH, Idar Oberstein, Germany).

The composition of the CEM I 42.5 R cement was determined with three different methods: (1) a combination of wet chemistry, microwave plasma atomic emission spectrometry (MP-AES) and Carbon Sulfur Determinator (CSD) according to EN 196-2 and EN 451-1, (2) X-ray fluorescence spectroscopy (XRF) according to EN 196-2 and (3) quantitative X-ray diffraction analysis (QXRD) using the Rietveld method. The results are compared in Table 1, and Table 2 shows the mineral composition of the employed silica fume.

### 2.2. Experiments

All samples were prepared by adding in each case 12.5 g of freshly ground hardened cement paste (either with or without silica fume) to 250 mL of diluted acetic acid with systematically varied concentrations of acetic acid (AcH) per gram of hydrated cement paste (hcp). This resulted in 24 samples of suspension with different acid concentrations between 0 and 60.2 mmol/g_(*hcp*)_ in the case of PC and 36 samples of suspension with acid concentrations between 0 and 308 mmol/g_(*hcp*)_ in the case of PC + SF. These samples were shaken at room temperature in closed bottles in a Hei-MIX Reax 2 overhead shaker until thermodynamic equilibrium was reached (101 days for PC and 146 days for PC + SF), which was controlled by periodically measuring the pH value of the suspensions using a Seven Multi pH meter (Mettler-Toledo GmbH, Giessen, Germany) until it was constant. Afterward, the suspensions were filtered using 60 mL of deionized water to wash the bottle and filter. Both the original solution and the washing water were combined for chemical analysis, and the mass was determined. The thereby obtained solids on the filter were dried overnight at 40 °C, stored in a desiccator over silica gel at room temperature until the mass was constant, and the original suspensions, as well as the filtered liquid and solid fractions, were photographed to compare the colors.

The concentrations of the following metal ions were determined in the solutions after filtration by means of microwave plasma atomic emission spectrometry (MP-AES) with a 4100 by Agilent Technologies (Santa Clara, CA, USA) using cesium as the calibration standard: Na, Mg, Al, Si, K, Ca and Fe.

The chemical composition of the solids was determined by X-ray fluorescence spectroscopy (XRF, EDX 8000 by Shimadzu, Kyoto, Japan) according to EN 196-2 after measuring the loss of ignition. Powder X-ray diffraction (XRD) was performed with a D8 Advance (Bruker AXS Advanced X-ray Solutions GmbH, Karlsruhe, Germany) equipped with a Lynxeye Detector (5° opening angle) utilizing Copper K_α_ radiation in a 2θ range between 5 and 70°. Rietveld evaluation was performed for quantitative analysis (QXRD). Background scattering was subtracted using Bruker software EVA 13.0.0.2. Simultaneous thermal analysis (STA) was performed with an STA PT 1600 (Linseis Messgeräte GmbH, Selb, Germany) on approx. 50 mg in a temperature range between 25 and 1000 °C with a heating rate of 10 K/min under a nitrogen atmosphere in a corundum crucible. Total (closed) porosity was determined after oven drying at 105 °C for 16 h by Mercury Intrusion Porosimetry (MIP) using an AutoPore V (Micromeritics GmbH, Unterschleißheim, Germany) with a maximum injection pressure of 400 MPa. An Eltra CS 2000 Carbon Sulfur Determinator (CSD, Eltra, Haan, Germany) was used to quantify the sulfide and carbonatic CO_2_ content in the dry cement.

### 2.3. Modeling

To model the attack of acetic acid on hardened cement paste with and without silica fume, IPHREEQC was used in Matlab. For this purpose, first, an instance of IPHREEQC was created in Matlab via the actxserver command, and the thermodynamic database “CEMDATA18” [16] was called (constants for acetate salts and complexes were introduced as well [3,8]). The input for the IPHREEQC instance is entered within a string cell, which can then be executed via a function. The modeling was divided into two steps. In the first step, the hydration is simulated, which means that water is mixed with the solid components of cement and allowed to react until reaching equilibrium. In the second step, the cement paste, which is the output of the first step, is placed in solutions with the same acid concentrations as in the experiments. The modeled results of the dissolution reaction are then compared with those of the experimental dissolution tests.

The composition of the cement and silica fume was entered as input for the hydration reaction, as determined by wet chemistry, MP-AES and CSD according to EN 196-2 and EN 451-1 (see Table 1 column 1 and Table 2). The mass of water was fixed at 1 kg, which means that the amount of material for the individual clinker minerals was calculated from the material composition of the cement based on the specified water/cement ratio of 0.40. This slightly higher *w*/*c* ratio in the model (compared to 0.35 in experiments) is more realistic than the experiment using the hardened cement paste because it can further hydrate during the curing in the water before being ground and added into the acid solutions. Additionally, the milling eliminates the effects of different capillary porosities, which would be the consequence of varying *w*/*c* ratios in larger specimens. In addition, no significant differences in the modeled composition of the hardened cement paste were observed (see Section 3). After completion of the hydration reactions, the various hydration products are used for further calculations before being added as input parameters in the second step of the model. The CSH phases are modeled as a solid solution according to the CSH3T model [17]. Another solid solution (Al-Fe siliceous hydrogarnet [18]) was used for the phases containing aluminum and iron. The porosity of the hydrated cement paste, $p_{initial}$, was then calculated via Formula (1) by comparing the simulated solid volumes after hydration with those of the starting materials,

(1) $$p_{\mathrm{initial}}=\frac{\left(V_{\mathrm{paste}}-V_{\mathrm{solids}}\right)}{V_{\mathrm{paste}}}$$

where:

- The volume of the paste is $V_{\mathrm{paste}\;}=\left(V_{\mathrm{water}}{+V}_{\mathrm{cement}}\right)\cdot x$;
- The volume of hydrated solids is $V_{\mathrm{solids}}=\sum^{\;}{(n}_{\mathrm{solid},i}{\cdot V}_{\mathrm{molar},i})$.

When transferring the output from step 1 as input for step 2, conversions are necessary. In the laboratory tests, 12.5 g of hardened cement paste is added to 250 mL of solution. In Formula (2), the factor *x* is calculated to adjust the substance quantities to this ratio. It also considers the scaling of 50 g cement paste in a 1000 mL solution as follows,

(2) $$x=\frac{50}{\left(w+c\right)}$$

where:

- *w* is the mass of water in g;
- *c* is the mass of cement in g.

This factor is then used in Formula (3) to calculate the quantity of the individual solid phases,

(3) $$n_{\mathrm{solid}}{=n}_{\mathrm{hydrated}}\cdot x$$

where:

- $n_{\mathrm{hydrated}}$ is the quantity of one hydration product in mol;
- x is the factor for 50 g hydrated paste in 1 kg solution.

If acetic acid in the form of acetate is now added to IPHREEQC, the volume of the solution increases to more than 1000 mL since the water content is fixed at 1 kg. This solution volume, which increases proportionally to the acid content, is taken into account via the factor *y* in Formula (4),

(4) $$y=\frac{{(V}_{\mathrm{water}}{+n}_{\mathrm{acetate}}{\cdot V}_{m,\mathrm{acetate}})}{1000\;\mathrm{mL}}$$

where:

- $V_{\mathrm{water}}$ is the volume of water in the solution → fixed to 1 kgw ≈ 1000 mL;
- $n_{\mathrm{acetate}}$ is the quantity of acetate in the solution in mol;
- $V_{m,\mathrm{acetate}}$ is the molar volume of acetate in cm^3^/mol.

The factor *y* is then used in Formula (5) to adjust the input content $n_{input,i,j}$ of the solid phase components of the hydrated cement paste to the increasing volume of acid solution,

(5) $$n_{\mathrm{input},i,j}{=n}_{\mathrm{solid},i}{\cdot y}_{j}$$

where:

- $n_{\mathrm{input},\;i,j}$ is the quantity of solid i at acid concentration step *j* in mol;
- $n_{\mathrm{solid},\;i}$ is the quantity of hydration product i in mol (output from Step 1);
- y is the factor for increased solution volume.

After performing the dissolution calculations, the material quantities of the products must be formatted for comparison with the experimental results. For this purpose, the material quantities are calculated back via the factor *y* to the hardened cement paste mass of 12.5 g per 250 mL acid solution added in the laboratory test.

The volume fractions of the individual solids $f_{V,\mathrm{solid},I,j}$ are calculated on the basis of the quantities obtained with the molar volume of the solids, as shown in Formula (6),

(6) $$f_{V,\mathrm{solid},i,j}=\frac{n_{\mathrm{out},\mathrm{solid},i,j}{\cdot V}_{m,i}}{\sum^{\;}{(n}_{\mathrm{solid},i}{\cdot V}_{m,i}{)\cdot (1+p}_{\mathrm{initial}})\cdot y\;}$$

where:

- $n_{\mathrm{out},\mathrm{solid},i,j}$ is the quantity of solid i at acid concentration step *j* in mol;
- $V_{m,i}$ is the molar volume of solid i in cm^3^/mol;
- $n_{\mathrm{solid},i}$ is the quantity of hydration product i in mol (output from Step 1);
- $p_{\mathrm{initial}}$ is the initial porosity of hydrated cement paste input;
- y is the factor for increased solution volume.

The mass fraction of Portlandite was calculated from the material quantities using Formula (7) to compare the modeled Portlandite content in the cement paste remaining after an acid attack with the results of the STA tests on the same cement paste residues,

(7) $$f_{m,\mathrm{solid},i,j}=\frac{n_{\mathrm{out},\mathrm{solid},i,j}{\cdot M}_{i}}{\sum^{\;}{{(n}_{\mathrm{out},\mathrm{solid},i}{\cdot M}_{i})}_{j}\;}$$

where:

- $n_{\mathrm{out},\mathrm{solid},i,j}$ is the quantity of solid i at acid concentration step *j* in mol;
- $M_{i}$ is the molar mass of solid i in g/mol.

The mass fractions of the oxides were calculated with Formula (8) to compare the oxide composition (CaO, SiO_2_, Al_2_O_3_ and Fe_2_O_3_) in the remaining solid from modeled results with the data from XRF analysis,

(8) $$f_{m,\mathrm{oxide},i,j}=\underset{j}{\sum }\left(\frac{n_{\mathrm{out},\mathrm{solid},i,j}}{\sum^{\;}n_{\mathrm{out},\mathrm{solid},i,j}}\cdot \frac{\nu_{oxide,i,j}\cdot M_{oxide}}{M_{i}}\right)\;$$

where:

- $n_{\mathrm{out},\mathrm{solid},i,j}$ is the quantity of solid i at acid concentration step *j* in mol;
- $\nu_{\mathrm{oxide},i,j}$ is the stoichiometric coefficient of oxide components of the solid phase i at acid concentration step j in mol;
- $M_{\mathrm{oxide}}$ is the molar mass of oxide in g/mol;
- $M_{i}$ is the molar mass of solid phase i in g/mol.

## 3. Results

Before the final input parameters for the model were set, different configurations were tested. It was shown that, as a result of different *w*/*c* ratios (0.35, 0.40 and 1.00), no significant differences in the modeled composition of the hardened cement paste were observed after the first model step (hydration). For this reason, the *w*/*c* ratio could be set to 0.40, which is slightly higher compared with 0.35 in the experimental part. As described earlier, this *w*/*c* ratio is more realistic as the hardened cement paste (mixed with a *w*/*c* ratio of 0.35) can further hydrate during the water storage until the age of 28 days. Differences in (capillary) porosity of the hardened cement paste have no influence since the samples were finely ground before being added to the acid solutions, and the dosage was weight-based.

Aside from the different *w*/*c* ratios, three different options for the input of the cement composition were tested too. Input implementation for the cement composition via phases from the database according to the composition assessed by QXRD analysis and via XRF results, as well as the chemical composition of oxides determined by wet chemistry, MP-AES and CSD according to EN 196-2 and EN 451-1, showed no significant differences. However, the input by means of the oxides determined according to the standards seemed more accurate than both XRF and QXRD results, which was the reason for selecting this option. Modeling results of the phase compositions (in vol. %) of the hydrated cement paste from the hydration step (Model 1) are listed in Table 3 for the mixture consisting purely of Portland cement (PC) and the mixture with Portland cement and 11% Silica Fume (PC + SF).

**Figure 1:**
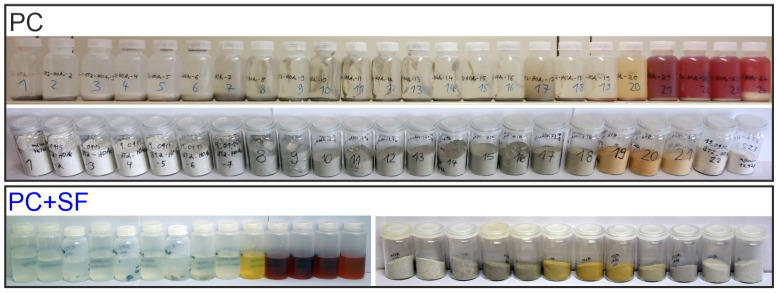
Photographs of selected suspensions before filtration and of the solids after filtration for PC, as well as some of the liquid and solid fractions after filtration for PC + SF (for each series with decreasing pH from left to right).

After pH equilibrium was reached, the samples showed different colors both for the liquid and solid fractions, as shown in Figure 1 for selected samples of PC and PC + SF. For the liquid fraction of PC, three different colorations can be observed: colorless in the pH range of 12.6 to 6.4, light brown around 5.8 and, finally, red between 5.3 and 3.8. The solids even show four colors: light grey between pH 12.6 and 11.3, dark grey between 11.0 and 6.6, light brown between 6.4 and 5.3 followed by light grey from 4.8 to 3.8. For PC + SF, the liquid phase was colorless between pH 12.8 and 6.3, yellow around 5.5, dark red between 4.8 and 3.6 and pale red around 2.6. The related solids exhibited the following colorations: light grey between 12.8 and 11.4, dark grey between 11.1 and 10.3, light brown between 9.5 and 5.5, brown grey around 4.8, dark grey around 4.2 and light grey between 3.6 and 2.6.

The intense reddening of the liquid phases for low pH values is probably due to leached iron, which reacts with acetic acid to form red ferric acetate aqueous complex and further hydrolyzes to reddish brown ferric hydroxide colloid, which is very soluble [19]. The colors of the solids are more difficult to interpret because they may be influenced by colored compounds subsequently precipitated during filtration due to the small amount of water used for washing (see the discussion concerning XRD during drying).

**Figure 2:**
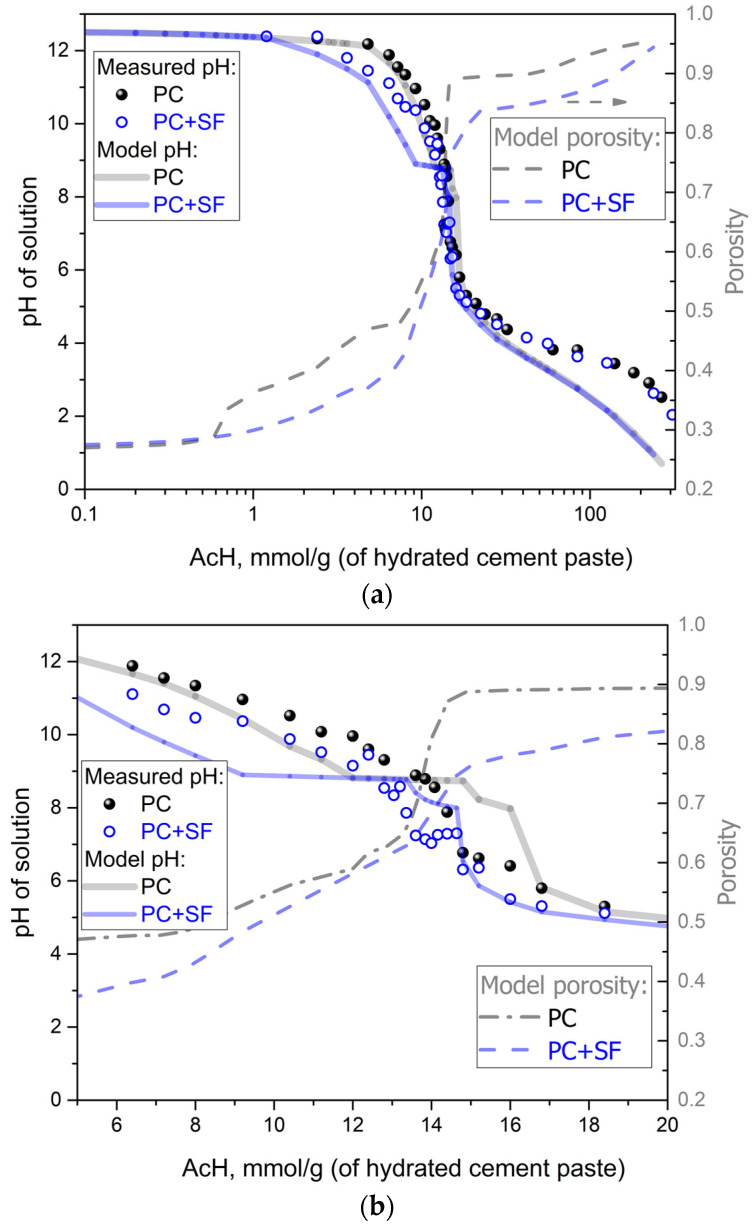

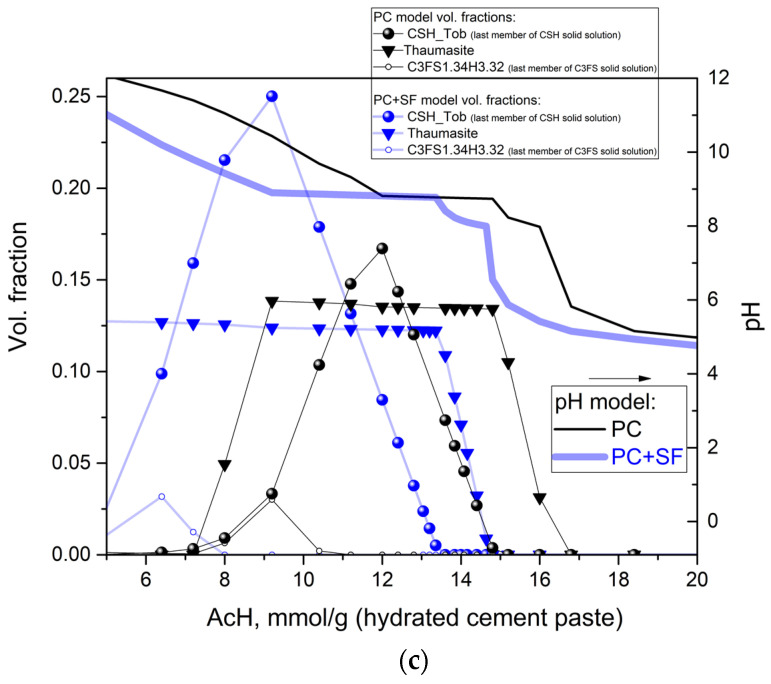
Change of pH as a function of acid concentration: full range of acid concentration (**a**) and zoom-in inset (**b**). The shape of the predicted pH is related to the dissolution (**c**), indicating a decrease in the vol. fraction of solid solutions and Thaumasite.

The pH of the suspensions after equilibrium is reached is a good indicator for the dissolution processes that have occurred at different acid concentrations. In Figure 2a,b, the experimentally measured and the modeled pH is shown in combination with the modeled porosity for different acid concentrations. Figure 2a shows an overview of the complete range of acid concentrations tested for both mixtures of PC and PC + SF. At acid concentrations below a few mmol of AcH per g hcp, the pH stays almost constant with a value higher than 12 for both mixtures as a result of the buffering effect of Portlandite. With increasing acid concentrations, the pH drops progressively, from gradual to abrupt changes. The pH of the pure PC mixtures starts to drop at higher acid concentrations than for the PC + SF mixture due to a higher content of Portlandite and has, therefore, a stronger buffering effect. The largest drop in the pH value occurs for acid concentrations between 5 and 20 mmol/g_(*hcp*)_. The resulting pH values and porosities for this range of acid concentrations are shown in detail in Figure 2b. For both mixtures, a plateau appears at a pH value of around nine in the modeled curves correlating to a buffering effect. In Figure 2c, this range of pH values matches the incongruent dissolution (mostly decalcification) of Tobermorite, which is the component of the CSH solid solution with the lowest Ca/Si ratio (see Figure 2c).

The plateau in the PC + SF curve starts at lower acid concentrations and includes a wider range compared with the plateau in the PC curve. The earlier start of the plateau in the PC + SF curve is again resulting from the lower content of Portlandite, whereas the longer plateau correlates to the higher content of CSH phases (and also the Tobermorite end member) due to the pozzolanic reaction of the silica fume with Portlandite during the (initial, step 1) cement hydration. The measured pH curves from the experiments do not show an obvious plateau as the modeled curves do, indicating a limitation of the thermodynamic model (discussed in Section 4). Adjacent to the first plateau, there is a second smaller plateau in the modeled pH curves correlating to the dissolution of Thaumasite (see Figure 2c). Further increasing acid concentrations led to a relatively fast drop in pH until the curves started to flatten out for acid concentrations higher than 20 mmol/g_(*hcp*)_. For acid concentrations this high, the model predicts slightly lower pH values than the measurements, indicating the limitation of the used thermodynamic database for low pH regions. Despite the observed discrepancies, in total, the modeled curves are in good agreement with the measured pH values, as no fitting parameter was used in this study.

The modeled porosity for both mixtures is also shown in these figures to illustrate the connection between dissolution reactions and pH change. The biggest increase in porosity takes place in the same range of acid concentrations as the biggest drop in pH. This area also fits the area where most dissolution reactions take place. The porosity of the PC mixtures increases earlier (i.e., at lower acid concentrations) due to the higher content of Portlandite and its dissolution. Furthermore, after Portlandite dissolution, the increase in porosity has a steep incline in the area of the pH plateau related to the Tobermorite dissolution. After the depletion of Ca-based phases, the change in porosity is very small compared to the dissolution of amorphous silica. The porosities of the PC + SF mixtures show a slower increase at lower acid concentrations due to the lower content of Portlandite available for dissolution. With further increase in acid concentrations, the higher content of CSH phases (Tobermorite) and, after its decalcification, amorphous silica in the PC + SF mixture results in a gradual approach of the PC + SF curve to the PC curve (the difference at 20 mmol AcH still being more than 5 vol.%). Dissolution processes naturally include an increase in dissolved ions in the acid solution.

**Figure 3:**
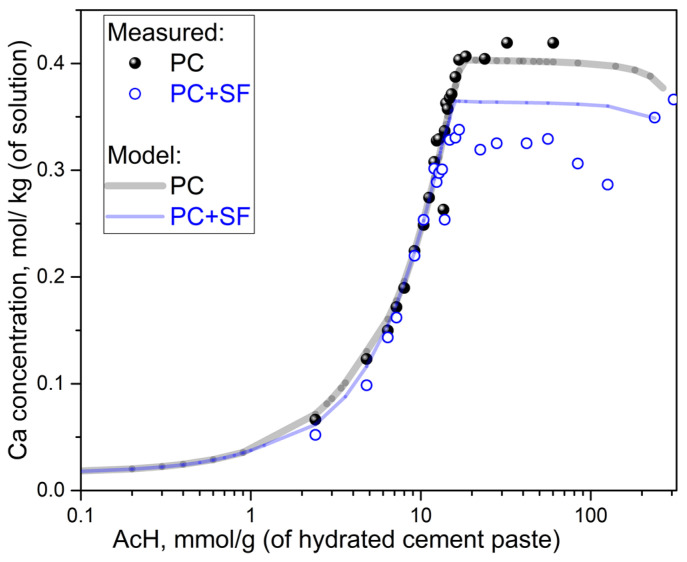
Concentration of dissolved calcium as a function of acid concentration, both for PC and PC + SF.

**Figure 4:**
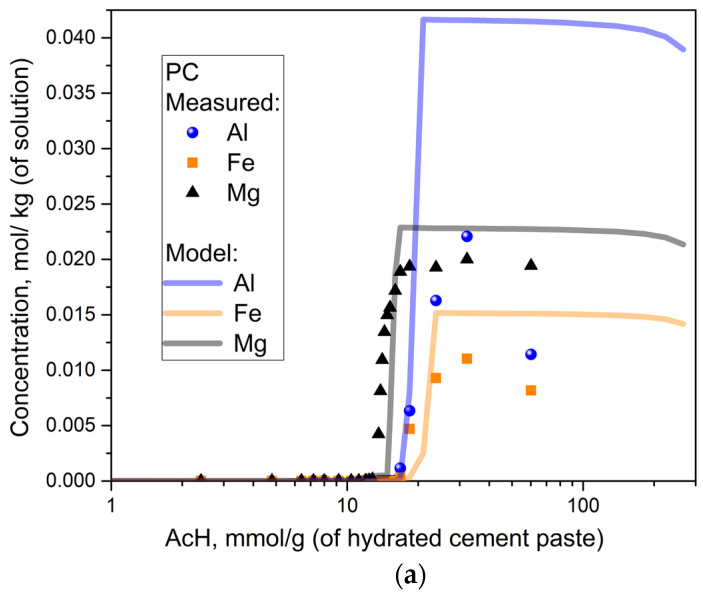

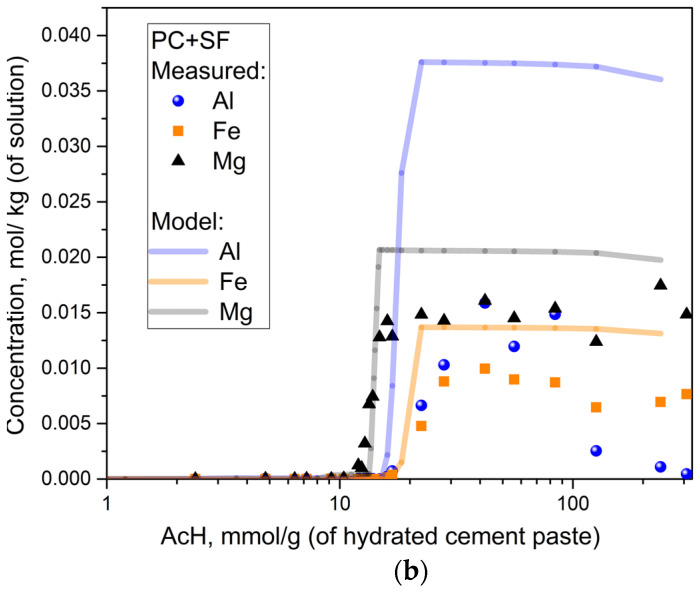
Concentration of dissolved (Al, Fe and Mg) elements as a function of acid concentration: PC (**a**) and PC + SF samples (**b**).

**Figure 5:**
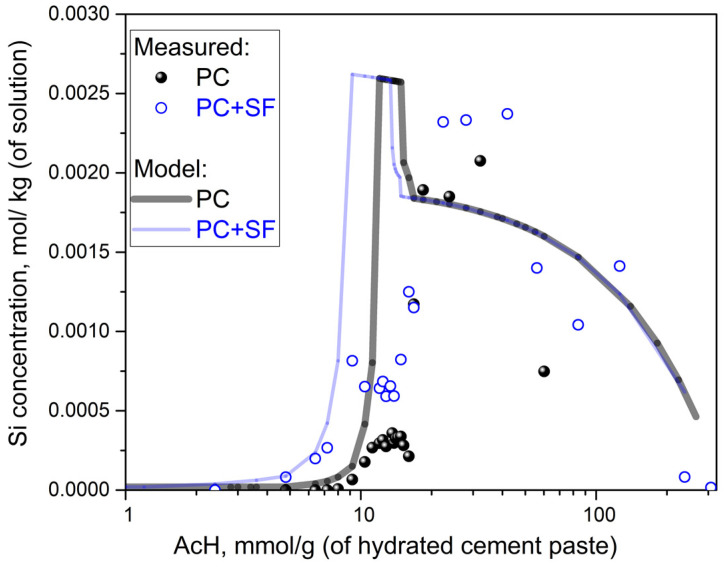
Concentration of dissolved Si as a function of acid concentration: comparison of PC vs. PC + SF paste powder samples.

Figure 3, Figure 4 and Figure 5 show measured and modeled ion concentrations in the acid solutions for both PC and PC + SF mixtures and different acid concentrations. Figure 3 shows the measured and modeled concentrations of calcium ions in different acid solutions. The initial incline of the curves is very similar for both mixtures and modeled as well as measured concentrations. Only at higher acid concentrations does the “final” plateau differs between the two mixtures, being higher for PC than for PC + SF, which is expected due to the respectively higher total content of calcium, which forms more aqueous Ca–acetate complexes. The comparison between the model and experiment for both mixtures shows small differences at higher acid concentrations. For the PC mixtures, the differences are very small, and due to a limited range of experimental data, it cannot be verified if the slight underestimation would also be present at acid concentrations higher than 60.2 mmol/g_(*hcp*)._ The deviations are more pronounced for PC + SF than PC, where the data also shows more scattering. The plateau of the modeled PC + SF curve is slightly higher than the measured values, from approximately 12 to 126 mmol/g_(*hcp*)_. However, the measured calcium contents at the highest acid concentrations (238 and 308 mmol/g_(*hcp*)_) seem to match the modeled values.

Figure 4a,b show the amount of completely dissolved aluminum, iron and magnesium ions in solutions with PC or PC + SF and different acid concentrations. Each curve shows a characteristic threshold value, after which the initial very low amount of dissolved ions (for low acid concentrations) is followed by a relatively steep increase of dissolved ions in a small range of acid concentrations, leading to an almost constant plateau at the maximum concentration. The model captures the range of acid concentration with the biggest increase of dissolved ions in good agreement with measurements for all ions (Al, Fe and Mg) and both mixtures. However, the model overestimates the plateau of maximum ion concentration, indicating that more ions are being dissolved in the model compared to the experiment. The difference is particularly pronounced for aluminum ions. As the initial content of ions contained in the hydrated cement paste is equal for the model and experiment, this difference could be explained both by model uncertainty for low pH Al chemistry and/or measurement artifacts (precipitation of Ca- and Al-acetates during filtration). Experimental artifacts observed in measurement results (XRD, ion concentrations) are further discussed later.

Figure 5 shows the concentration of total dissolved silicon ions for both PC and PC + SF at different acid concentrations. The modeled curves for the two mixtures show a similar progression with matching parts at low and high acid concentrations. At medium acid concentrations, compared with PC, the PC + SF (model and measurement) curves shift towards lower concentrations (similar to Ca and pH results). Both PC and PC + SF exhibit similar shapes with a steep incline followed by a short plateau before the curves drop. The PC + SF mixture shows an incline starting at lower concentrations due to the lower content of Portlandite compared to the PC mixture, which results in an earlier dissolution of CSH phases, including silicon ions. The relatively short ‘one step’ peak in the model curve is due to the lack of a more detailed description of the CSH (Tobermorite end-member) incongruent dissolution, which, according to the experiments, occurs more gradually and shifts towards lower pH values. The almost constant plateau after the ‘one step’ peak is wider for PC + SF as a result of the higher content of CSH phases or, in general, silicon ions in the initial hydrated cement paste compared to the PC mix without silica fume. The experimentally measured values seem to have a differing shape of the curve with almost no dissolved silicon ions for low acid concentrations. The increase in ion concentrations with increasing acid concentrations, including a small plateau at lower silicon levels, in the measured curve is not as steep as it is for the modeled curve. After the low plateau, the ion concentrations increase to a higher plateau over a wider range of acid concentrations compared to the modeled curves. The measured values show a decrease of dissolved ions for high acid concentrations correlating to the model. Thus, the model predicted the difference well between the PC and PC + SF mixtures regarding the onset and initial part of the increase in the concentration of dissolved ions, which are in good agreement with the measurements.

**Figure 6:**
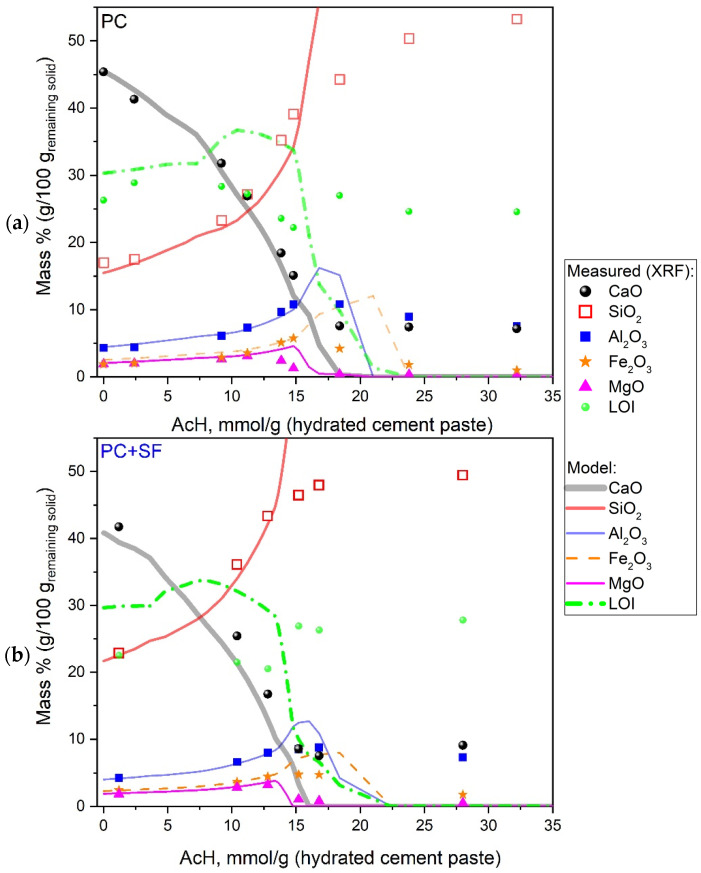
Concentration of undissolved elements in remaining solids as a function of acid concentration: comparison of XRF measurements with model predictions: PC (**a**) and PC + SF samples (**b**).

In addition to the analysis of the acid solution, the composition of the remaining solids was investigated after reaching equilibrium. Figure 6a,b shows the comparison of the composition of oxides in the remaining solids obtained by XRF measurement and model predictions. The main oxides CaO, SiO_2_, Al_2_O_3_, Fe_2_O_3_ and MgO are to be complemented by the loss of ignition (LOI). LOI consists mainly of water (H_2_O) and carbon dioxide (CO_2_) that are initially bound to the remaining (degraded) solid samples and released during the calcination treatment procedure before XRF measurement. However, the comparison of the model and experiment was challenging in the low pH range, mainly due to the model assumptions for precipitation of anhydrous SiO_2_, which in reality has an unknown (relatively high) amount of water content. More details, also including other challenges in modeling and experimental artifacts, are discussed in Section 4. For the PC mixture, Figure 6a shows good agreement in chemical compositions of the remaining solids between the experiment and the model, only at lower acid concentrations up to around 15 mmol/g_(*hcp*)_. When exceeding this acid concentration, the model LOI and all oxide elements, except for SiO_2_, exhibit a fast drop to zero, while amorphous SiO_2_ becomes the only solid phase rapidly reaching 100 wt. %. In the measured data points, this systematic change in LOI is not visible. Here the oxides remain more or less constant at acid concentrations higher than 15 mmol/g_(*hcp*)_.

The PC + SF mixture in Figure 6b shows a similar progression of up to around 13 mmol/g_(*hcp*)_, which is slightly lower than pure PC; the modeled curves match the measured oxide composition. At higher concentrations, the LOI and the oxides, except for SiO_2_, drop to zero, while SiO_2_ reaches 100 wt. %. Again, the measured oxide fractions remain almost constant for higher acid concentrations.

**Figure 7:**
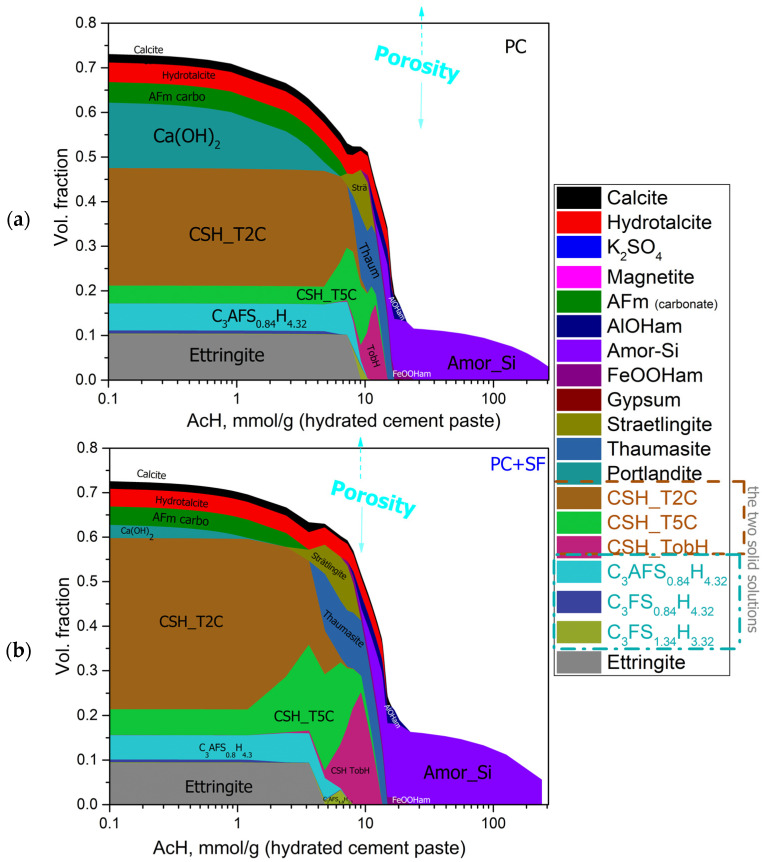
Predicted volume fractions (Formula (6)) and porosity (Formula (1)) as a function of acid concentration in PC (**a**) and PC + SF (**b**). Phase volumes are obtained from phase concentrations in remaining solids and their molar volumes, while fractions are related to the volume of the initial bulk paste calculated from the *w*/*c* ratio and their unhydrated densities (Formula (1)).

Aside from the chemical composition, the model also predicted the phase (mineralogical) composition of the remaining solids. Such modeling results are shown in Figure 7a,b as volume fractions in relation to the initially added hydrated cement paste. In this way, the increase in total porosity with increasing acid concentration due to dissolution processes was quantified.

The differences obtained due to the SF addition match the differences already shown in the above figures. The most obvious difference is the bigger fraction of Portlandite in the mixture without silica fume. In PC + SF, the proportion of Portlandite is reduced due to the pozzolanic reaction with the amorphous SiO_2_ from the silica fume. The volume fraction of CSH phases is increased compared with the PC mixture as a result of this reaction. The higher content of Portlandite and the resulting increased OH buffering at low acid concentrations leads to the beginning of the decalcification of CSH phases and the dissolution of other shifted phases towards higher acid concentrations for the PC samples, as compared with the PC + SF samples. The lower content of CSH phases in the PC samples leads to a narrower range of acid concentrations at which the (incongruent) dissolution takes place. For both mixtures at acid concentrations around 22 mmol/g_(*hcp*),_ the remaining solids consist purely of amorphous silica (SiO_2_), whose water content was not captured by the model.

As the amount of Portlandite has a large effect on the dissolution behavior of the samples at low acid concentrations, the Portlandite content of the initial input and the remaining solids was measured via STA for selected acid concentrations in the lower range. The results from STA measurements of PC and PC + SF samples are listed together with the corresponding values obtained from the model in Table 4. The measured values for the initial powder samples without leaching in water are lower than the modeled ones, which can be explained by the incomplete hydration after 28 days in the experiment. The relation shifts to higher Portlandite content in the measurements when acid is introduced due to the incomplete dissolution reactions compared to the model. However, the values after an acid attack, and especially the acid concentrations at which Portlandite is dissolved, show completely good agreement between the measurements and model.

**Figure 8:**
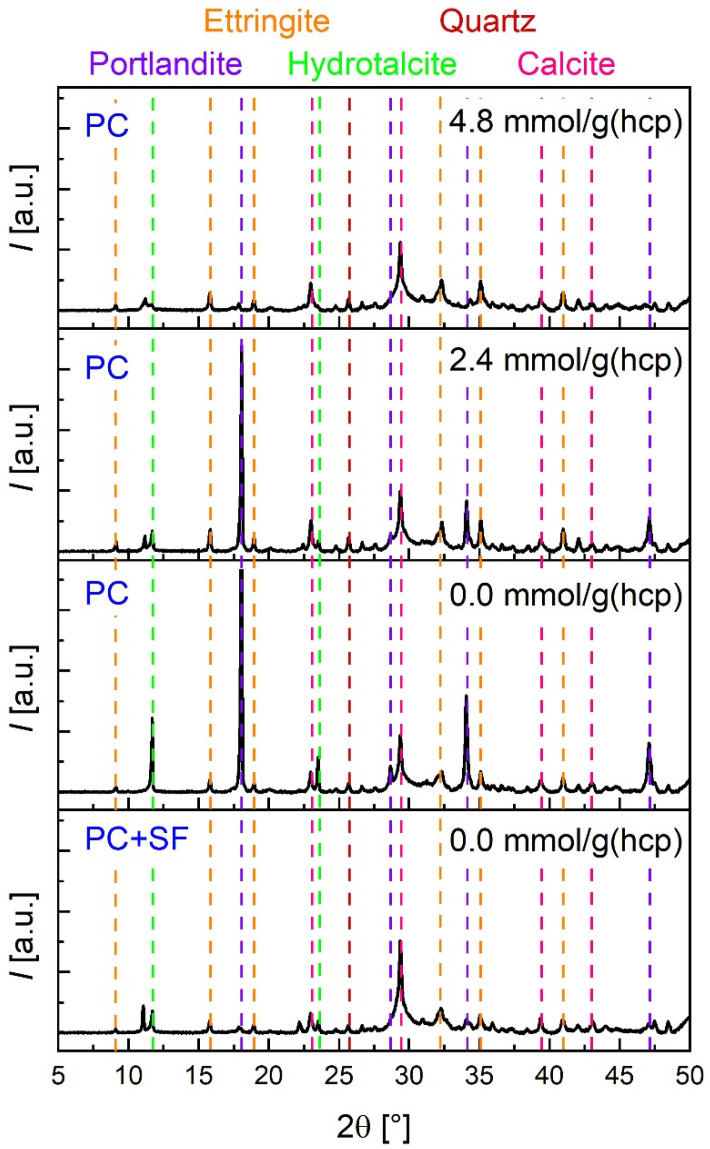
X-ray diffraction patterns of hardened cement paste with and without silica fume after leaching with water and acetic acid. Portlandite is only detectable for the sample without silica fume for the concentrations 0.0 and 2.4 mmol/g_(*hcp*)_.

After filtration, the dried solid fractions were investigated by means of XRD. Figure 8 shows parts of the obtained patterns of hardened cement paste with and without silica fume after leaching with water and with two low concentrations of acetic acid, respectively. In all four samples, Ettringite, Hydrotalcite, Calcite and a small amount of Quartz were found. Portlandite was only detectable for the samples without silica fume with acid concentrations of zero and in a lower quantity of 2.4 mmol/g_(*hcp*)_. These observed phases are also in good agreement with the phases predicted by the model (Figure 7). All predicted crystalline phases were found except for the very small amount of K_2_SO_4_. The observed decreased fraction of Portlandite is comparable to the finding in the STA measurements and the prediction of the model. Although, according to the model and STA measurements, small amounts of Portlandite should additionally be present for the hardened cement paste with silica fume at 0 mmol/g_(*hcp*)_ and for the one without silica fume at 4.8 mmol/g_(*hcp*)_. Due to the considerable amount of background scattering, these small fractions could not be determined with XRD.

The filtrate obtained by filtration was used to characterize the ions in the solution in an attempt to ensure that the composition was related to the ones in the solids. Furthermore, only a small and constant amount of water was used for washing in order to prevent the additional dissolution of solids, which should be in equilibrium and remain part of the solid fraction. However, it was observed that with this experimental procedure, the filtration and washing process has a considerable influence on the results of the composition of the solid fraction.

**Figure 9:**
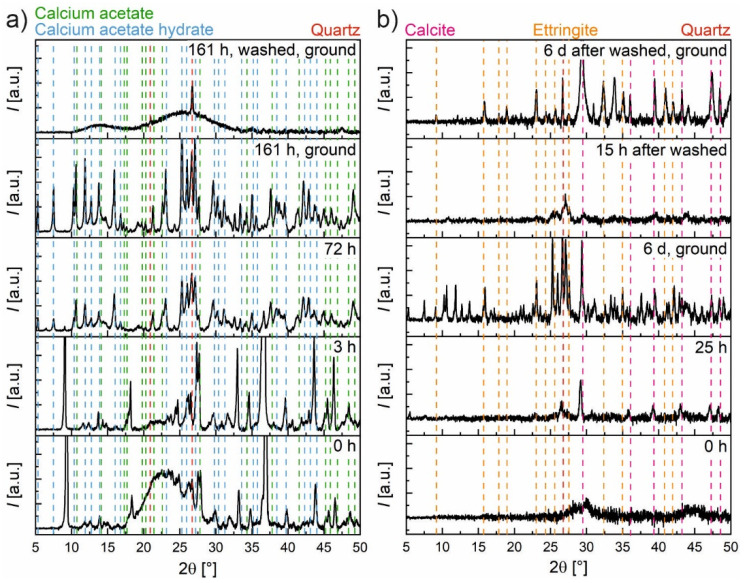
X-ray diffraction patterns of (**a**) one sample of PC after equilibration with diluted acetic acid (350 mmol/g_(*hcp*)_) during drying for 161 h and after thoroughly washing with demin. water (phase assignment for 161 h, ground), and of (**b**) one sample of PC + SF after equilibration with diluted acetic acid (11.2 mmol/g_(*hcp*)_) during drying for 6 d and of a parallel sample after thoroughly washing with demin. water (phase assignment for 6 d after washed, ground). Note that the diffractograms of the samples that were not ground may be slightly shifted in 2θ due to the improvised preparation in the sample holder.

This can be seen in Figure 9a, where XRD measurements were conducted at different times for a sample without silica fume initially in equilibration with diluted acetic acid (350 mmol/g_(*hcp*)_). Immediately after the wet sludge is removed from the storage tanks, despite the background correction, a pronounced amorphous scattering is visible in addition to the peaks of the crystalline phases. After three hours of drying in the XRD sample holder, this amorphous scattering almost vanished. After a total of about 72 h of drying at room conditions in this XRD sample holder, some peaks disappeared while some new ones appeared, mainly calcium acetate and calcium acetate hydrates. No significant changes took place thereafter in the investigated time period until 161 h. As expected, grounding this sample results in more distinct peaks, which mostly vanish after washing with an extensive amount of water. In the end, Quartz mainly remains. Only the phases of the last sample are in agreement with the known chemistry of altered hardened cement paste and the model prediction. Without thorough washing, different acetates (mainly calcium acetates and calcium acetate hydrates, in other samples also aluminum acetate hydroxide and Gypsum) are always present, depending on the water content.

In Figure 9b, a sample of PC + SF with a much lower acid concentration (11.2 mmol/g_(*hcp*)_) is shown. The wet sludge (0 h) shows no significant peaks. Drying this wet sludge for 25 h led to more pronounced peaks (mainly Calcite). After drying for 6 d and subsequent grounding, more peaks appeared (mostly calcium acetate hydrates), which now became better detectable due to the improved signal-to-noise ratio. A parallel sample was washed with 360 mL of water immediately after sampling and first measured after 15 h of drying and again after 6 d of drying in a desiccator over silica gel as well as subsequent grounding. It can be seen that washing resulted in fewer peaks than in the dried sludge, mainly Ettringite, Calcite and Quartz. If calcium and aluminum acetate (hydrates) are still present, they were only in traces. Thereby, it appears that washing the initial 12.5 g hardened cement paste with 360 mL of water is sufficient to prevent artifacts in the composition of the solids by subsequent precipitation. However, the model predicts, in contradiction to these observations, the presence of Hydrotalcite, Straetlingite and Thaumasite and the absence of Ettringite (see Figure 7b). It indeed seems to be the case that the ‘sample 6 d, ground’ contains less or no Ettringite and instead some Thaumasite (most peaks can be attributed to calcium acetate and calcium acetate hydrate, Figure 7a). Therefore, washing, on the one hand, prevented the enrichment of the solid phase with subsequently precipitated acetate (hydrates) or removed them, but, on the other hand, it had an influence on the phase composition probably by changing ion concentrations and pH values and thus the stability of phases.

The solids investigated in this study via XRF represent a similar state as the XRD pattern after ‘161 h, ground’ in Figure 9a, whereas XRD patterns in other studies [1,3,6,19,20,21,22] investigated bulk samples, which are more comparable to the example after ‘161 h, washed, ground’ in this figure.

Therefore, it can be concluded that the amount of calcium in the solids determined in this study tends to be too high, whereas the calcium in the solution tends to be too low. This error due to the filtration and washing process is expected to be negligible for low concentrations of acetic acid (there are almost no acetates and acetate hydrates detectable in Figure 8) and to increase with an increasing concentration of acetic acid and, therefore, the increasing presence of acetate ions (see Figure 6).

After these considerations, the question arises of which experimental procedure is most suitable for investigating the composition of the solids and solution in the equilibrium state. We suggest using finely ground hardened cement paste instead of bulk specimens if the information on the phases and concentrations at thermodynamic equilibrium or on the kinetics of the reactions involved are to be gained. In the case of bulk samples, they will probably develop different zones with different chemical compositions of the solids due to the influence of diffusion. Thus, the solution will not be in equilibrium with only a single state of altered cement paste. Investigating the composition of the solution can be performed by pipetting a small and defined volume before filtration, which ensures that the actual equilibrium state of the solution is investigated. Only then should the filtration be carried out, which should be accompanied by extensive washing. The amount of water should be sufficient to dissolute all acetates. Here it would be good to measure the combined volume of solution and also to determine the composition of this combined solution, which should be comparable to the result prior to the filtration process. After this, the composition of the solids should be determined. Nevertheless, determining the exact composition of the solids is much more challenging than for the solution since the washing procedure can lead to dissolution, which then has to be accounted for by calculation. This allows for determining the realistic chemical composition of the solids, although it should be stressed that the actual phase composition can hardly be determined since the investigations by means of XRD cannot be carried out in situ in equilibrium with the surrounding solution, and, therefore, the phase inventory can be changed by sampling and preparation.

## 4. Discussion

In this paper, the thermodynamic modeling of acidification and leaching of a realistic cement system was conducted in the newly developed Matlab code that links IPHREEQC with a recent TD database for cement hydration [16] and acetic acid solution [3,8]. The leaching and acidification cycle consisted of closely simulating batch experimental setups by adjusting the aggressive solution to hydrated solids ratios for thermodynamic re-equilibration calculations. Therefore, numerous batch experiments with hardened cement paste powder were conducted to determine the composition in equilibrium over a wide range of acid concentrations and pH.

Validation of the TD model is the first fundamental step in modeling acid attack of cementitious materials, as the much more challenging reactive-transport models [5,6] should couple the chemistry, i.e., reaction thermodynamics and kinetics [8,9,10] coupled with the transport phenomena. The main link between chemistry and transport is mostly (over) simplified by using an (apparent) diffusion coefficient that should consider the changes in porosity [11]. Typically, the acidic acid attack on cement-based materials is investigated by using large samples such as cylinders or cubes [1,3,6,19,20,21,22]. Therefore, the changes in chemical composition and structural and mechanical properties can be studied. However, if one is specifically interested in the chemical composition in thermodynamic equilibrium, this experimental setup has disadvantages. First, chemical reactions are influenced by the rate of diffusion of the ions from the surface of the samples to the inner parts or vice versa. Second, there can be solely one state of thermodynamic equilibrium investigated for a given combination of sample size and volume of the solution, and this is the case when the whole sample has reacted. Dyer [3] employed an inverse modeling approach to (empirically) estimate the apparent diffusion coefficient based on the measured pH increase of the exposure (external) solution. However, such an estimate of the apparent diffusion coefficients does not consider the time nor space dependency of the microstructural changes (dominantly porosity). It represents an overall diffusion rate throughout the whole specimen, both the degraded (decalcified) and sound (unaffected) cement paste, without considering the changes in porosity and diffusion coefficients throughout the different layers. Thus, first, the thermodynamic description needs to be well understood by adopting the equilibration of powdered cement in acidic solutions of varying concentrations, which represent fundamental inputs to tackle the transport and kinetic effects in the bulk samples.

**Figure 10:**
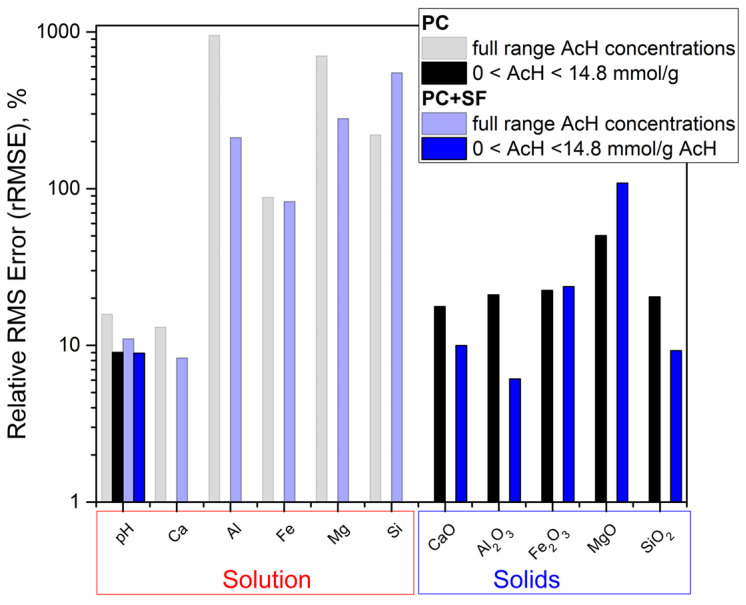
Deviations between model and experiment, calculated as relative root square mean error (rRSME) for pH, dissolved ions in acid solution and oxide composition in remaining solids, both for a full range of acid concentrations that range up to 14.8 mmol/g_(*hcp*)_.

During the acid attack of cementitious materials, the pH of the pore solution decreases, increasing the solubility equilibrium concentrations, e.g., of calcium, silicon and aluminum ions in the cement paste matrix, according to Le Chatelier’s principle of equilibrium law. This is because, during the acid attack, the ingress of protons removes OH^−^ from the equilibrium by forming water molecules (water dissociation constant). Likewise, organic acid anions, e.g., the acetate focused in this study, form aqueous complexes, namely metal-acetates, resulting in more dissolution of the corresponding solids. The main objective of this study is to validate the equilibrium results of the model by comparison with the results of the batch reaction experiments specially designed and performed in the current paper to reach this objective. Despite the observed discrepancies, we argue that the model curves are in good overall agreement with measurement values for the equilibrium concentrations of the dissolved ions (Figure 2, Figure 3, Figure 4 and Figure 5) and undissolved solids (Figure 6 and Figure 7). Figure 10 shows the deviations between the model and experiment calculated as the relative root square mean error (rRSME).

For the ion concentrations in the solution, the total deviations were calculated for each ion over the full range of acid concentrations, whereas the deviations between oxide compositions of the remaining solids obtained from XRD measurements and modeling were only calculated for lower acid concentrations up to 14.8 mmol/g_(*hcp*)_. For higher acid concentrations, the discussed challenges in modeling the LOI made comparison impossible. The deviation of the pH curves is calculated for the full range as well as for the lower range of acid concentrations. To objectively judge the agreement, one should also consider that all model parameters are fundamentally and independently chosen based on chemical composition and the thermodynamic database, and no ‘fitting’ calibration was adapted throughout the whole study.

The sequential dissolution of the phases, Portlandite -> AFm -> CSH -> AFt was in agreement with previous findings for Ca leaching [2,3,23] and is followed by the dissolution of Straetlingite and Thaumasite, which is in agreement with our XRD data. The model was not fully able to predict a gradual smooth decrease of the pH as observed by the measured curves (Figure 2, Figure 3, Figure 4 and Figure 5). Instead, an obvious plateau indicated a limitation of the thermodynamic model, primarily related to the chosen CSH3T solid solution model. This discrepancy was most obvious when predicting Si concentrations in solution, which still correctly predicts the onset but happens in a too-short pH range, with a one-step-like peak overestimate of the measurement (Figure 5). This is directly related to the limitation of the CSH3T (Tobermorite end-member) incongruent dissolution, which in reality occurs more gradually, involving the dissolution of more intermediate phases over a wider pH range. For a better agreement with measurements, a more detailed CSH model is needed to capture the incongruent nature of the CSH dissolution even better. The discrete solid solution CSH model proposed by Walker et al. [24] would be a way to improve predictions (pH, Ca and Si concentrations, as well as more realistic changes in molar volume) that are suitable for implementation in IPHREEQC. However, such a model lacks a description of known CSH structural features and uptake of other elements (e.g., alkalies, aluminum, etc.). To capture both solubility and structural features, Lothenbach et al. [16] recommended the use of recent truly multi-site mixing models for CSH, but they are still the subject of ongoing research. Moreover, such advanced models are not possible to implement in the used version of IPHREEQC software(Version 3.6.2-15100).

Aside from improvements in modeling CSH solubility, the highest discrepancy observed for the dissolved aluminum ions could not only be attributed to the lack of better model parameters but also to the measurement artifacts, such as precipitation of calcium acetate hydrate and AlOH(acetate)_2_ during filtration. The lack of an adequate thermodynamic description would primarily be related to the uncertainties in modeling precipitation of different types of aluminum hydroxide gels/crystals and/or AlOH(acetate)_2_ precipitation and complexation reactions [3]. Improvement of model predictions would require either lower solubility of aluminum precipitates or less stable Al complexation (i.e., lower formation constants).

Regarding the composition of the remaining solids, significant discrepancies between the model and measurements were obtained only in the low pH range, which involved the precipitation of anhydrous SiO_2_. This is mainly due to the model assumptions for LOI predictions which do not include the unknown amount of water content in the silica gel, while the type of aluminate and iron precipitates are questionable. Namely, aside from the selected AlOHam and FeOOHam, precipitation of other gel phases could be expected as well as AlOH(acetate)_2_ and FeOH(acetate)_2_. The LOI predictions are based only on water, CO_2_ and sulfate release from mineral hydrates (no acetate salts), while no water was assigned to the anhydrous SiO_2_ (Amor_Sl). Thus, the LOI of the remaining solids is particularly difficult to predict for the very low pH range. This is because the carbon and water release in the samples may originate from the acetate salts, carbonation and presence of unknown water amounts in silica-based and other (Fe- and Al-based) gels. In particular, we highlight here the artifacts experimentally observed (XRD results) related to the precipitations (e.g., acetate salts) occurring during the filtration process.

## 5. Conclusions

From the results of this study, the following conclusions can be drawn:

(1) The 11% replacement of cement by silica fume (SF) reduced the Portlandite content from 22 to 6.7, assuming a complete pozzolanic reaction in the model, resulting in higher concentrations of CSH phases. Initial deviations from measured Portlandite indicate an incomplete hydration reaction. The predicted depletion of Portlandite in acidic conditions was in good agreement with the measurements;
(2) This change in mineral composition led to an earlier decrease of pH due to less buffering effect of Portlandite and lower overall Ca content;
(3) The applied modeling approach shows an overall good agreement with experiments, resulting in a relative root square mean error of below 10% for pH and Ca (in solution and remaining solids) without applying any fitting parameters;
(4) The discrepancies were higher after the depletion of primary hydrated phases at very high acid concentrations, which related to uncertainties of the model in predicting secondary Al-, Si- and Fe-gel precipitates;
(5) The discrepancy from list number four, at high acid concentrations (equilibrium pH < 4), could also be explained by challenges in sample preparation, especially the observed artifacts due to drying and/or precipitation (primarily Ca- and Al-acetate salts) during the filtration of the remaining solids after acid exposures;
(6) Based on the observations made in this study, experimental challenges are discussed, and an experimental procedure is proposed to investigate the equilibrium state and minimize experimental artifacts;

The foundation for improved reactive transport models was laid with the findings of this study regarding the thermodynamic modeling of hydrated cement paste dissolved by acetic acid as an example for acids which form highly soluble salts. In future research, we plan to extend the model by including the effects of transport verified by experiments on molded specimens of hardened cement paste, as well as different effects of aggregates. Such reactive transport models will improve the prediction of the service life of different concrete mixtures exposed to acid attack.

## Figures and Tables

**Table 1:** Physical properties and mineralogical composition of the employed CEM I 42.5 R cement.

Determined by Wet Chemistry, MP-AES and CSD		Determined by XRF		Determined by QXRD	
Property	Mass [%]	Property	Mass [%]	Property	Mass [%]
Na_2_O	0.13	Na_2_O	0.10	Na_2_O	0.15
MgO	2.47	MgO	2.36	MgO	2.24
Al_2_O_3_	5.70	Al_2_O_3_	5.48	Al_2_O_3_	5.68
SiO_2_ (total)	19.85	SiO_2_	19.71	SiO_2_	19.4
K_2_O	1.72	K_2_O	1.50	K_2_O	1.21
CaO	60.68	CaO	59.62	CaO	62.88
MnO	0.08	MnO	0.07		
Fe_2_O_3_	3.25	Fe_2_O_3_	3.12	Fe_2_O_3_	3.19
Loss of ignition (corrected)	2.01	Loss of ignition	2.01		
Insoluble residue	0.92				
SO_3_	3.72			SO_3_	3.47
S^2−^	0.02				
Cl^−^	0.08				
CaO (free)	2.37				
CO_2_ (carbonatic)	1.07			CO_2_	1.49
		P_2_O_5_	0.16		
		TiO_2_	0.24	TiO_2_	0.00
				H_2_O	0.61

**Table 2:** Mineralogical composition of the employed silica fume.

Determined by Wet Chemistry and MP-AES	
Property	Mass [%]
Na_2_O	0.20
SiO_2_ (total)	94.12
K_2_O	0.94
Loss of ignition (corrected)	1.11
SO_3_	0.09
Cl^−^	0.06
CaO (free)	0.0

**Table 3:** Composition of the hydrated cement paste from Model 1 (including initial porosity compared to the experimental determined by MIP).

Phase	PC [vol. %]	PC + SF [vol. %]
Calcite	1.94	1.80
Ettringite	10.19	9.17
Hydrotalcite	4.39	4.11
K_2_SO_4_	0.00	0.12
AFm (carbonate)	4.16	4.02
Portlandite	16.77	5.30
Al-Fe siliceous hydrogarnet (solid solution) C_3_A_0.50_F_0.50_S_0.84_H_4.32_	6.54	6.14
C_3_FS_0.84_H_4.32_	0.49	0.45
C_3_FS_1.34_H_4.32_	0.11	0.10
CSH3T (solid solution) T2C (Ca/Si = 1.5: C_1.5_S_1_H_2.5_)	26.22	39.61
T5C (Ca/Si = 1.0: C_1.25_S_1.25_H_2.5_)	3.96	5.98
TobH (Ca/Si = 0.67: C_1_S_1.5_H_2.5_)	0.02	0.03
Initial porosity Experimental by MIP	25.21 18.6	23.16 19.9

**Table 4:** Comparison of Portlandite fractions determined by modeling and by STA experiments.

Acid Concentration [mmol/g_(*hcp*)_]	Mass Loss Due to Portlandite, Measured with STA [%]	Calculated Fraction of Portlandite from STA [%]	Mass Fraction of Portlandite from Model [%]
Initial (PC)	2.5	10.3	22.01
0 (PC)	2.3	9.5	19.98
2.4 (PC)	1.4	5.8	3.42
4.8 (PC)	0.1	0.4	0.45
Initial (PC + SF)	1.0	4.1	6.70
0 (PC + SF)	0.3	1.2	4.28
1.0 (PC + SF)	0.1	0.4	0.51

## Data Availability

The data relevant to this study is available on request from the corresponding author.
